# Idiopathic partial central diabetes insipidus

**DOI:** 10.31744/einstein_journal/2023RC0124

**Published:** 2023-01-27

**Authors:** Larissa Fabre, Viviane Calice da Silva

**Affiliations:** 1 Hospital Regional Hans Dieter Schmidt Joinville SC Brazil Hospital Regional Hans Dieter Schmidt, Joinville, SC, Brazil.

**Keywords:** Diabetes insipidus, Hypernatremia, Deamino arginine vasopressin, Polyuria, Water

## Abstract

Diabetes insipidus is a rare disorder characterized by the inability to concentrate urine, which results in hypotonic urine and increased urinary volume. It may occur because of antidiuretic hormone deficiency or resistance to its action in the renal tubules. When there is a deficiency in the synthesis of antidiuretic hormones, diabetes insipidus is called central; when there is resistance to its action in the renal tubules, it is said to be nephrogenic. We report a case of idiopathic partial central diabetes insipidus and highlight the management and treatment of the disease.

## INTRODUCTION

Diabetes insipidus is a rare disorder characterized by the inability to concentrate urine. There are two types: central diabetes insipidus (CDI), which results from antidiuretic hormone deficiency, and nephrogenic diabetes insipidus (NDI), which is characterized by antidiuretic hormone resistance in the renal tubules.^(1)^

In this report, we describe a case of idiopathic partial CDI in a patient treated at a public hospital in southern Brazil and review the literature on the management and treatment of this disease.

## CASE REPORT

A 39-year-old male was admitted to the emergency room in February 2021 for acute diarrhea, poor general condition, and tremors. The patient was previously independent in daily activities but had a history of schizophrenia since he was 14 years old and of chronic polydipsia. Until hospital admission, his renal function had been preserved. His recent serum creatinine was within the normal range for his age group, and a recent ultrasound of the kidneys and urinary tract was also normal.

His medications included biperiden (2mg 3 tablets/day), chlorpromazine (100mg, 4 tablets/day), haloperidol (5mg, 3 tablets/day), and lithium carbonate (300mg, 4 tablets/day). He did not recall the duration of his lithium carbonate use. Physical examination upon admission revealed tachycardia and decreased consciousness (Glasgow Coma Scale [GCS] = 10). Complementary tests showed acute kidney injury (creatinine: 3.6mg/dL, urea: 128mg/dL, sodium: 142mmol/L) and lithium intoxication (lithium level: 4.5mEq/L). The patient’s status further deteriorated necessitating orotracheal intubation, renal replacement therapy, and transfer to the intensive care unit (ICU).

During his ICU stay, he showed a persistent hypernatremia and polyuria, requiring intravenous infusion of glucose 5% and hydration with 0.9% normal saline. After a few weeks, the patient was discharged from the ICU.

In the ward, the patient showed partial recovery of renal function allowing the discontinuation of renal replacement therapy. The patient developed hypotension, fever, hypernatremia, and polyuria. The patient’s urine output was approximately 9L/day, with a sodium level of 177mEq/L, serum osmolality of 395mOsm/kg H₂O, and urinary osmolarity of 165mOsm/L. At this time, the patient was severely dehydrated and underwent volume expansion for 72 hours until volume reconstitution, resulting in a partial decrease in serum sodium levels, albeit still hypernatremic. Considering the hyposmolar polyuria with hypernatremia, a diagnosis of diabetes insipidus was made.

**Figure 1:**
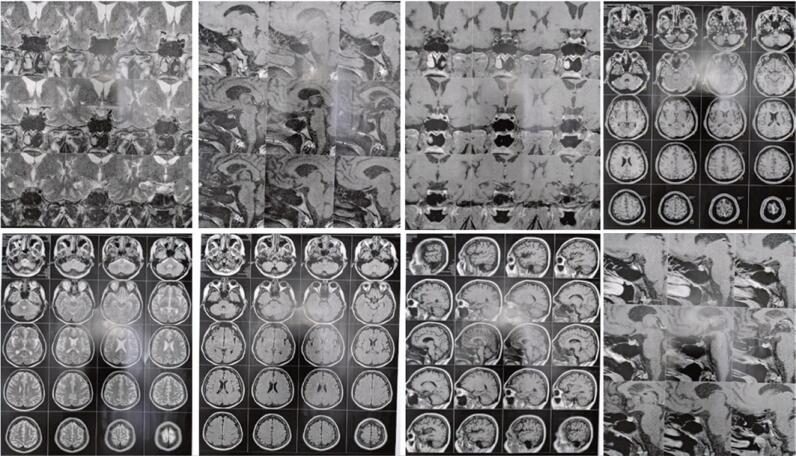
Nuclear magnetic resonance of the patient’s skull and pituitary

The investigation for diabetes insipidus was started with a cranial computed tomography scan, which showed normal results. Subsequently, cranial magnetic resonance imaging (MRI) was performed, which revealed normal brain parenchyma and the absence of the usual T1 hypersignal of the neurohypophysis (Figure 1). These MRI finding were compatible with the clinical suspicion of diabetes insipidus.

To determine whether the diabetes insipidus was central or nephrogenic, the patient received one spray of desmopressin in each nostril (10mcg/spray). The initial hypothesis was that the patient had NDI due to lithium intoxication; however, with the application of intranasal desmopressin, the serum osmolality decreased to 317.72mOsm/kg H₂O, the sodium level normalized, and the urinary osmolarity increased to 330mOsm/L, leading to the diagnosis of partial CDI.

During hospitalization in the ward, the patient developed multiple infections, which were treated with several courses of antibiotics (piperacillin/tazobactam, meropenem, vancomycin, and polymyxin B). In addition, he acquired COVID-19 with associated deep venous thrombosis. Important to note is that the incidence of deep venous thrombosis is higher in patients with hyperosmolality.^(2)^

In the context of COVID-19, the patient presented with secondary pulmonary fungal infections and received antifungal treatment (amphotericin B and fluconazole). Amphotericin B may impair the ability to concentrate urine by reducing the activity of medullary renal adenylate cyclase, thus reducing the effect of vasopressin on the collecting ducts.^(3)^ However, its use was necessary due to the occurrence of an invasive fungal infection.

The patient went into cardiorespiratory arrest but was revived, with return of spontaneous circulation. As a result of his cardiac arrest, the patient was reintubated, which necessitated changing his intranasal desmopressin to subcutaneous and intravenous formulations. After extubation, intranasal desmopressin was resumed, but the patient maintained urinary osmolarity approximately 330mOsm/L. We opted for the combination of carbamazepine and thiazide diuretics, as well as the the prescription of a low-sodium diet. With these, urinary osmolarity increased to 363mOsm/L, serum osmolality decreased to 295mOsm/kg H₂O, and sodium remained within the normal range.

**Figure 2:**
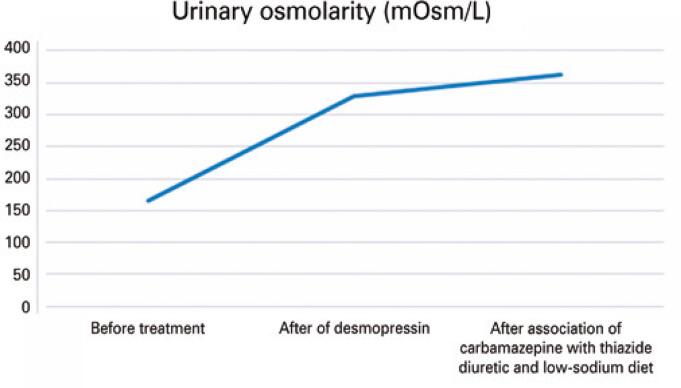
Urinary osmolarity over the course of treatment

**Figure 3 f03:**
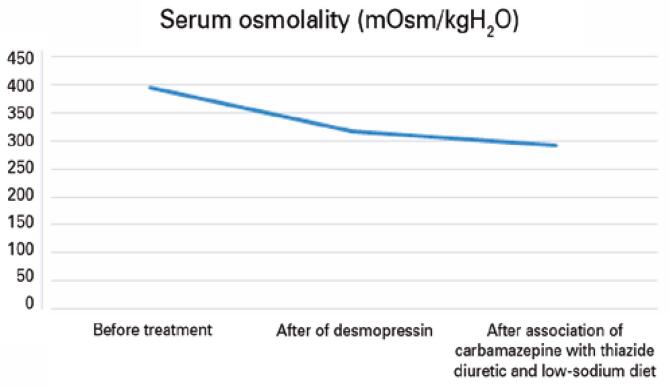
Serum osmolality over the course of treatment

**Figure 4 f04:**
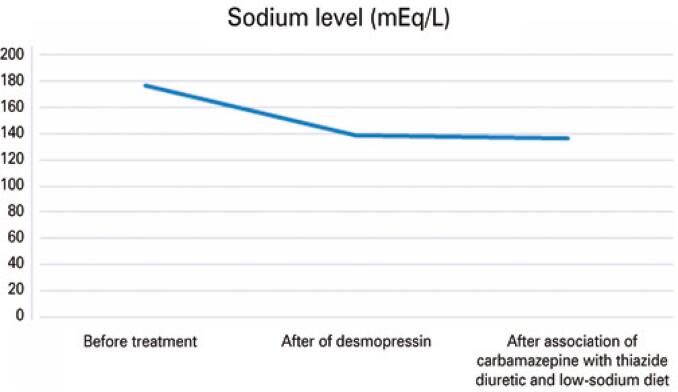
Sodium level over the course of treatment

**Figure 5 f05:**
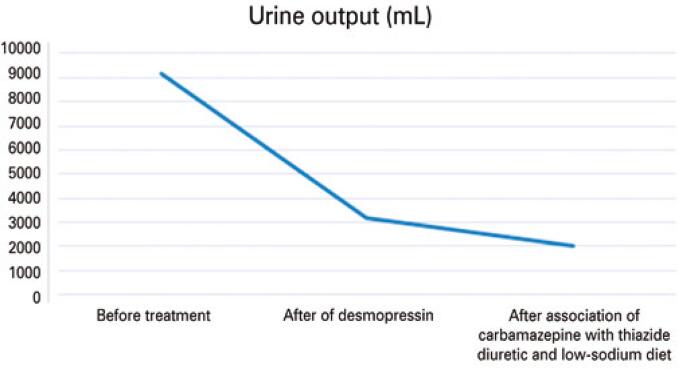
Urine output over the course of treatment

**Figure 6 f06:**
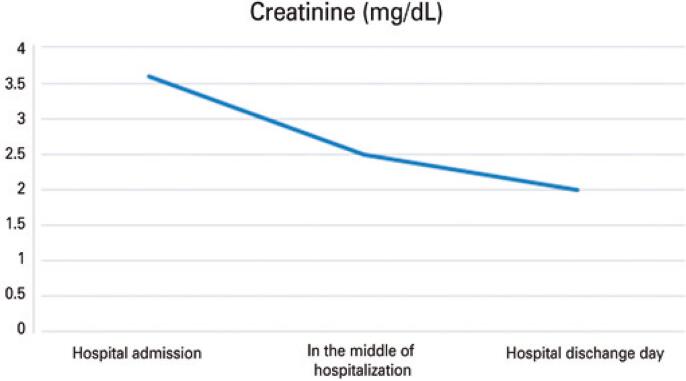
Creatinine level during hospitalization

**Figure 7 f07:**
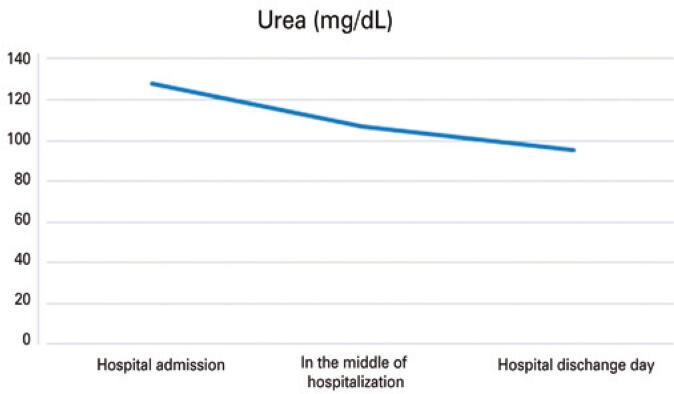
Urea level during hospitalization

The patient was discharged from the hospital in November 2021 with the following medications: carbamazepine 200mg 1 tablet/day, risperidone 1mg 1 tablet/night, biperiden 2mg 1 tablet/day, hydrochlorothiazide 25mg 1 tablet/day, and desmopressin 3 puffs in each nostril every 12 hours. Figures 2 to 7 [Fig f03] [Fig f04] [Fig f05] [Fig f06] [Fig f07] illustrate the profile of urinary osmolarity, serum osmolality, sodium level, urine output, creatinine, and urea during hospitalization and the evolution of the case with treatment.

On the day of discharge, the patient’s test results showed creatinine of 2.0mg/dL, urea of 95mg/dL, and sodium of 137mmol/L. Despite the persistence of changes in kidney function, renal biopsy was not performed during hospitalization due to the COVID-19 pandemic and restrictions on performing the procedures. Currently, the patient is being followed up at the Nephrology Outpatient Clinic, with stable renal function, stage 3b chronic kidney disease, and controlled sodium. This study was approved by the Research Ethics Committee of *Hospital Regional Hans Dieter Schmidt* under CAAE: 49143821.1.0000.5363; #4.882.486.

## DISCUSSION

### Etiology

Diabetes insipidus is generally diagnosed within the context of the differential diagnoses for polyuria. When polyuria is significant in a patient’s clinical presentation, it must be determined if it is due to an osmotic effect (urine osmolality >300mOsm/kg), whose main causes can be divided between organic and electrolyte, or if polyuria is hyposmolar (urine osmolality <200mOsm/kg).^(4)^

Organic polyuria may be induced by urea, glucose, or mannitol. Electrolytic polyuria can usually occur with imbalance of chloride, bicarbonate, or ketoanions, as seen in Bartter syndrome, renal tubular acidosis, or diabetic ketoacidosis.^(4)^

Hyposmolar polyuria occurs as a result of psychogenic polydipsia or diabetes insipidus. The diagnosis of diabetes insipidus is made if the patient has serum hypernatremia in the background of hyposmolar urine. The psychogenic polydipsia, which is characterized by excessive water intake, will never cause serum hypernatremia.^(4)^

The main causes of CDI and NDI are reported in table 1.^(5)^

**Table 1:** Etiology of diabetes insipidus

Type of diabetes insipidus	Etiology
Acquired central diabetes insipidus	- Trauma (surgery, deceleration injury) - Neoplastic (craniopharyngeoma, meningioma, germinoma, metastases) - Vascular (cerebral/hypothalamic hemorrhage, infarction or ligation of anterior communicating artery aneurysma) - Granulomatous (histiocytosis, sarcoidosis) - Infectious (meningitis, encephalitis, tuberculosis, toxoplasmosis, HIV infection) - Inflammatory/autoimmune (lymphocytic infundibuloneurohypophysitis, IgG4 neurohypophysitis) - Idiopathic
Congenital central diabetes insipidus	*- AVP* gene mutation - Wolfram syndrome (*WFS1* gene mutation) - Central diabetes insipidus in context of malabsorptive diarrhea (*PCSK1* gene)
Acquired nephrogenic diabetes insipidus	- Drug exposure (lithium, demeclocyclin, cisplatin, etc.) - Hypokalemia - Hypercalcemia - Infiltrating lesions (sarcoidosis, amyloidosis, multiple myeloma) - Sickle cell anemia
Congenital nephrogenic diabetes insipidus	- X-linked *AVPR2* gene mutations - Autosomal recessive or dominant *AQP2* gene mutations

*AVP*: arginine vasopressin; *AVPR2*: arginine vasopressin receptor 2; *AQP2*: aquaporin 2.

Central diabetes insipidus, which is characterized by antidiuretic hormone (ADH) deficiency, occurs because of disturbances in the sites involved in hormone secretion.^(6)^ Cranial MRI can help determine the cause.^(1)^

The most common causes of CDI are idiopathic, primary or secondary tumors, neurosurgery, and trauma.^(7)^ Approximately 30% of cases are idiopathic.^(8)^ In the present case, the precise etiology for the disorder could not be identified, despite the cranial MRI.

### Clinical manifestations and diagnosis

The main symptoms of CDI are polyuria, nocturia, and polydipsia due to abnormalities in the ability to concentrate urine.^(9)^ In CDI there is a high 24-hour urine output of >50mL/kg body weight, with associated polydipsia of more than 3L/day.^(10)^ In patients with polyuria, a plasma sodium concentration above 142mEq/L suggests a diagnosis of diabetes insipidus, as opposed to a concentration below 137mEq/L.^(11)^

Plasma sodium between 137mEq/L and 142mEq/L can be seen in both patients with diabetes insipidus and primary polydipsia. In both conditions, a fluid restriction test may be necessary.^(11)^ The purpose of the test is to increase plasma osmolality to above 295mOsm/kg or plasma sodium to above 145mEq/L and to assess the response to desmopressin administration.^(11)^ For patients with a baseline plasma osmolality above 295mOsm/kg or plasma sodium above 145mEq/L, as with the current case, the water restriction test may be dispensed by assessment of urinary osmolality and the response to desmopressin administration.^(1)^ Following the administration of desmopressin, an increase of >100% in urine osmolality characterizes complete CDI.^(4)^ When there osmolality increases by <10%, a diagnosis of NDI is made.^(4)^ Intermediate increases in urinary osmolality in response to desmopressin administration may occur due to either partial CDI or partial NDI.^(12)^ In these patients, the evaluation of not only the percentage variation but also the absolute numbers can help in the distinction. Patients with partial CDI usually achieve urinary osmolality >300mOsm/kg after desmopressin administration, as in the case presented, whereas patients with NDI typically have persistently diluted urine.^(13)^

Data from patients’ clinical histories can also help in this differentiation. In adults, the onset of symptoms is usually sudden in cases of CDI and insidious in cases of NDI.^(13)^

Fenske et al. aimed to increase the diagnostic accuracy of the fluid restriction test by combining it with copeptin measurement.^(14)^ Copeptin is the C-terminal segment of the arginine vasopressin (AVP) precursor peptide and can be easily measured, mirroring AVP concentrations.^(15)^ In a cohort of 50 patients with polyuria-polydipsia syndrome, baseline plasma copeptin levels >20pmol/L characterized patients with NDI, while levels <2.6pmoL/L after an overnight fluid restriction test indicated CDI.^(14)^For the present case, the copeptin measurement was unavailable at the hospital and therefore, could not be measured.

Patients with CDI often have normal-to-high serum sodium concentrations. Cases of moderate to severe hypernatremia can occur when thirst is diminished or cannot be expressed.^(8)^ In this case, when the patient was deprived of free access to water while on mechanical ventilation in ICU, he started to present with hypernatremia.

### Treatment

There are three main options for treating CDI. Desmopressin is an ADH analog and the medication of choice in almost all cases. It is available in liquid form, and is usually administered intranasally, or in the form of oral tablets and parenteral formulations.^(16)^Medications such as chlorpropamide, carbamazepine, and thiazide diuretics are also used.^(17,18)^ A low renal solute load formula is also started in order to reduce urinary water loss.^(19)^

The choice of therapy varies according to the severity of disorder.^(19)^ It is essential to maintain a stable fluid balance in managing patients with CDI. Serum sodium levels above 160mmol/L are associated with mortality in up to 75% of patients.^(3)^ Knowledge and correct management of diabetes insipidus are essential for the recovery of these patients.
